# Supplementary material for: Catalysis-dependent selenium incorporation and migration in the nitrogenase active site iron-molybdenum cofactor
Source: eLife. 2015 Dec 16;4:e11620. doi: 10.7554/eLife.11620 (PMC4755756; doi:10.7554/eLife.11620)
Supplement: Supplementary file 1. — (A) Quantification of selenium occupancies at FeMoSe-cofactor belt x2B, x5A and x3A positions. (B) Data collection and refinement statistics. DOI: http://dx.doi.org/10.7554/eLife.11620.013 [file elife-11620-supp1.docx]

**Supplementary file 1A.**

**Quantification of selenium occupancies at FeMoSe-cofactor belt x2B, x5A and x3A positions.** Se-occupancies were calculated from the anomalous difference density using the average anomalous Fe-density determined at 12662 eV as an internal standard (f’’ (Se) = 3.84 e; f’’ (Fe) = 1.50 e; f’’ (S) = 0.24 e), and subtracting the anomalous density contribution from the residual S. Occupancy errors were estimated to be 5% based on a comparison of density values for the two crystallographically independent copies of the FeMo-cofactor per Av1. The lower threshold for the minimum occupancy that can be detected is estimated as 2% based on the root mean square values of the anomalous electron density maps.

| **Structure** | **x2B** | **Selenium occupancy at position x [%]**  **x5A** | **x3A** |
| --- | --- | --- | --- |
| Av1-Se2B | 100 | 2 | 1 |
| Av1-Se-fq-2 | 89 | 9 | 4 |
| Av1-Se-fq-46  Av1-Se-fq-341  Av1-Se-fq-921  Av1-Se-fq-1785  Av1-Se-fq-2141  Av1-Se-fq-5361  Av1-Se-CO | 75  39  27  17  11  0.3  10 | 17  33  32  8  5  0.3  44 | 10  27  21  8  6  0.8  35 |

**Supplementary file 1B.**

**Data collection and refinement statistics.** Av1-Se2B represents the selectively Se2B-incorporated resting state of Av1. Av1-Se-CO represents the CO-bound form of Av1-Se2B. Structural data of Av1-Se2B obtained by freeze quenching (fq) the enzyme during turnover are named as follows: Av1-Se-fq-2, Av1-Se-fq-46, Av1-Se-fq-341, Av1-Se-fq-921, Av1-Se-fq-1785, Av1-Se-fq-2141, Av1-Se-fq-5361 (numbers represent C_2_H_4_ produced per active site). Values in brackets represent the highest resolution shell.

| **Data collection statistics** | **Av1-Se2B** | **Av1-Se-fq-2** |
| --- | --- | --- |
| Wavelength (Å) | 0.97918 | 0.97918 |
| Resolution range (Å) | 38.76 – 1.60 (1.69 – 1.60) | 39.64 – 1.50 (1.58 – 1.50) |
| Unique reflections | 256,394 (37,336) | 314,688 (46,280) |
| Completeness (%)  Anom. Completeness (%) | 97.8 (97.8)  96.5 (96.5) | 99.4 (99.2)  99.0 (98.3) |
| Multiplicity | 7.1 (7.1) | 7.0 (7.0) |
| Space group | P2_1_ | P2_1_ |
| Unit cell parameters |  |  |
| a, b, c | 77.58, 130.82, 107.13 | 77.31, 130.45, 107.15 |
| α, β, γ | 90.0, 108.85, 90.0 | 90.0, 108.92, 90.0 |
| R_merge_ | 0.151 (0.833) | 0.143 (0.759) |
| R_p.i.m._  CC_1/2_ | 0.064 (0.344)  0.996 (0.766) | 0.057 (0.308)  0.996 (0.779) |
| Mn (I / σ(I)) | 9.5 (2.3) | 8.2 (2.3) |
| **Data processing statistics** |  |  |
| R_cryst_ (%) | 15.17 | 14.18 |
| R_free_ (%) | 17.81 | 15.66 |
| r.m.s.d. bond lengths (Å) | 0.013 | 0.015 |
| r.m.s.d. bond angles (°) | 1.530 | 1.842 |
| Average B-factor (Å^2^)  Ramachandran: allowed (outliers) (%) | 8.27  99.57 (0.43) | 9.08  99.65 (0.35) |

| **Data collection statistics** | **Av1-Se-fq-46** | **Av1-Se-fq-341** |
| --- | --- | --- |
| Wavelength (Å) | 0.97918 | 0.97918 |
| Resolution range (Å) | 38.70 – 1.45 (1.53 – 1.45) | 38.65 – 1.32 (1.39 – 1.32) |
| Unique reflections | 345,205 (50,107) | 477,230 (69,613) |
| Completeness (%)  Anom. Completeness (%) | 94.5 (94.1)  94.3 (93.8) | 98.3 (98.3)  98.1 (97.9) |
| Multiplicity | 7.2 (7.1) | 6.9 (6.9) |
| Space group | P2_1_ | P2_1_ |
| Unit cell parameters |  |  |
| a, b, c | 81.14, 129.66, 107.20 | 80.95, 130.68, 106.91 |
| α, β, γ | 90.0, 110.56, 90.0 | 90.0, 110.59, 90.0 |
| R_merge_ | 0.105 (0.626) | 0.123 (0.767) |
| R_p.i.m._  CC_1/2_ | 0.042 (0.251)  0.998 (0.867) | 0.049 (0.311)  0.997 (0.777) |
| Mn (I / σ(I)) | 12.0 (3.1) | 9.4 (2.3) |
| **Data processing statistics** |  |  |
| R_cryst_ (%) | 15.15 | 13.88 |
| R_free_ (%) | 16.52 | 15.46 |
| r.m.s.d. bond lengths (Å) | 0.018 | 0.014 |
| r.m.s.d. bond angles (°) | 1.879 | 1.897 |
| Average B-factor (Å^2^)  Ramachandran: allowed (outliers) (%) | 6.97  99.45 (0.55) | 6.865  99.45 (0.55) |

| **Data collection statistics** | **Av1-Se-fq-921** | **Av1-Se-fq-1785** |
| --- | --- | --- |
| Wavelength (Å) | 0.97918 | 0.97918 |
| Resolution range (Å) | 38.99 – 1.64 (1.73 – 1.64) | 38.56 – 1.66 (1.75 – 1.66) |
| Unique reflections | 247,719 (36,011) | 239,196 (34,553) |
| Completeness (%)  Anom. Completeness (%) | 98.3 (98.0)  98.1 (97.8) | 98.4 (97.4)  98.2 (97.2) |
| Multiplicity | 7.0 (6.8) | 7.0 (6.7) |
| Space group | P2_1_ | P2_1_ |
| Unit cell parameters |  |  |
| a, b, c | 78.05, 132.03, 107.91 | 80.77, 130.33, 106.85 |
| α, β, γ | 90.0, 108.86, 90.0 | 90.0, 110.59, 90.0 |
| R_merge_ | 0.190 (0.796) | 0.134 (0.785) |
| R_p.i.m._  CC_1/2_ | 0.076 (0.326)  0.992 (0.769) | 0.054 (0.326)  0.996 (0.778) |
| Mn (I / σ(I)) | 6.5 (2.1) | 12.2 (2.8) |
| **Data processing statistics** |  |  |
| R_cryst_ (%) | 16.47 | 15.13 |
| R_free_ (%) | 19.11 | 18.08 |
| r.m.s.d. bond lengths (Å) | 0.019 | 0.018 |
| r.m.s.d. bond angles (°) | 1.942 | 1.870 |
| Average B-factor (Å^2^)  Ramachandran: allowed (outliers) (%) | 8.557  99.60 (0.40) | 7.81  99.65 (0.35) |

| **Data collection statistics** | **Av1-Se-fq-2141** | **Av1-Se-fq-5361** |
| --- | --- | --- |
| Wavelength (Å) | 0.97918 | 0.97918 |
| Resolution range (Å) | 38.63 – 1.65 (1.74 – 1.65) | 38.60 – 1.48 (1.56 – 1.48) |
| Unique reflections | 244,354 (35,162) | 315,907 (44,643) |
| Completeness (%)  Anom. Completeness (%) | 98.3 (97.2)  98.1 (96.9) | 95.3 (92.4)  94.6 (91.0) |
| Multiplicity | 6.9 (6.6) | 7.0 (6.7) |
| Space group | P2_1_ | P2_1_ |
| Unit cell parameters |  |  |
| a, b, c | 80.93, 130.27, 107.21 | 77.25, 130.31, 107.09 |
| α, β, γ | 90.0, 110.63, 90.0 | 90.0, 109.00, 90.0 |
| R_merge_ | 0.129 (0.808) | 0.147 (0.749) |
| R_p.i.m._  CC_1/2_ | 0.052 (0.337)  0.997 (0.817) | 0.059 (0.309)  0.995 (0.745) |
| Mn (I / σ(I)) | 10.8 (2.1) | 8.7 (2.4) |
| **Data processing statistics** |  |  |
| R_cryst_ (%) | 16.22 | 16.47 |
| R_free_ (%) | 19.33 | 18.04 |
| r.m.s.d. bond lengths (Å) | 0.019 | 0.018 |
| r.m.s.d. bond angles (°) | 1.882 | 1.941 |
| Average B-factor (Å^2^)  Ramachandran: allowed (outliers) (%) | 8.21  99.65 (0.35) | 7.17  99.70 (0.30) |

| **Data collection statistics** | **Av1-Se-CO** |
| --- | --- |
| Wavelength (Å) | 0.97918 |
| Resolution range (Å) | 38.71 – 1.53 (1.61 – 1.53) |
| Unique reflections | 301,848 (43,965) |
| Completeness (%)  Anom. Completeness (%) | 96.2 (96.1)  96.1 (95.9) |
| Multiplicity | 7.2 (7.2) |
| Space group | P2_1_ |
| Unit cell parameters |  |
| a, b, c | 81.07, 130.83, 107.32 |
| α, β, γ | 90.0, 110.64, 90.0 |
| R_merge_ | 0.127 (0.875) |
| R_p.i.m._  CC_1/2_ | 0.051 (0.345)  0.997 (0.737) |
| Mn (I / σ(I)) | 11.3 (2.1) |
| **Data processing statistics** |  |
| R_cryst_ (%) | 14.33 |
| R_free_ (%) | 16.52 |
| r.m.s.d. bond lengths (Å) | 0.015 |
| r.m.s.d. bond angles (°) | 1.855 |
| Average B-factor (Å^2^)  Ramachandran: allowed (outliers) (%) | 11.72  99.41 (0.59) |
